# Response of the Ubiquitous Pelagic Diatom *Thalassiosira weissflogii* to Darkness and Anoxia

**DOI:** 10.1371/journal.pone.0082605

**Published:** 2013-12-02

**Authors:** Anja Kamp, Peter Stief, Jan Knappe, Dirk de Beer

**Affiliations:** 1 Max Planck Institute for Marine Microbiology, Microsensor Group, Bremen, Germany; 2 Jacobs University Bremen, Molecular Life Science Research Center, Bremen, Germany; 3 University of Southern Denmark, Department of Biology, NordCEE, Odense, Denmark; Stazione Zoologica, Italy

## Abstract

*Thalassiosira weissflogii*, an abundant, nitrate-storing, bloom-forming diatom in the world’s oceans, can use its intracellular nitrate pool for dissimilatory nitrate reduction to ammonium (DNRA) after sudden shifts to darkness and anoxia, most likely as a survival mechanism. *T. weissflogii* cells that stored 4 mM ^15^N-nitrate consumed 1.15 (±0.25) fmol NO_3_^-^ cell^-1^ h^-1^ and simultaneously produced 1.57 (±0.21) fmol ^15^NH_4_^+^ cell^-1^ h^-1^ during the first 2 hours of dark/anoxic conditions. Ammonium produced from intracellular nitrate was excreted by the cells, indicating a dissimilatory rather than assimilatory pathway. Nitrite and the greenhouse gas nitrous oxide were produced at rates 2-3 orders of magnitude lower than the ammonium production rate. While DNRA activity was restricted to the first few hours of darkness and anoxia, the subsequent degradation of photopigments took weeks to months, supporting the earlier finding that diatoms resume photosynthesis even after extended exposure to darkness and anoxia. Considering the high global abundance of *T. weissflogii*, its production of ammonium and nitrous oxide might be of ecological importance for oceanic oxygen minimum zones and the atmosphere, respectively.

## Introduction

Diatoms are a key group of the eukaryotic phytoplankton of the world’s oceans from polar to tropical latitudes. Pelagic diatoms form massive phytoplankton blooms [1] and may sink to the seafloor in vast abundances [2]. Diatoms are responsible for 40% of the marine primary production, or 20% of the Earth’s primary production [3,4]. Thus, they play a key role in the oceanic C-cycle and their productivity supports large-scale coastal fisheries [5]. Diatoms can also survive for decades buried deep within the dark, O_2_-depleted sediment layers at the seafloor, where neither photosynthesis nor aerobic respiration is possible [6,7]. The survival mechanism under these non-phototrophic conditions is still poorly understood. Only recently, the dissimilatory use of NO_3_^-^ by the benthic diatom *Amphora coffeaeformis* was discovered as a possible survival mechanism in darkness and anoxia [8]. The study revealed that *A. coffeaeformis* stored NO_3_^-^ intracellularly and used it for Dissimilatory Nitrate Reduction to Ammonium (DNRA; NO_3_^-^ NO_2_^-^ NH_4_^+^) after sudden exposure to darkness and anoxia. Briefly, dissimilatory NO_3_^-^ reduction is an energy-generating pathway where NO_3_^-^ is taken as electron acceptor instead of O_2_ in respiratory processes. It preferentially occurs in environments in which O_2_ is scarce or in which steep O_2_ gradients exist. In the marine realm, coastal sediments, oceanic Oxygen Minimum Zones (OMZs), and suspended aggregates (“marine snow”) are prominent (micro)environments characterized by O_2_ shortage (e.g.[9-12]). Besides DNRA, denitrification (NO_3_^-^ NO_2_^-^ NO N_2_O N_2_) and anammox (oxidation of NH_4_^+^ to N_2_ with NO_2_^-^ as the electron acceptor) are important dissimilatory NO_3_^-^ reduction pathways. Dissimilatory NO_3_^-^ reduction has important implications for the marine N-cycle and is not least due to increasing use of synthetic fertilizers and subsequent pollution of rivers, estuaries, and coastal waters well studied (e.g. 13-17). However, our knowledge is almost exclusively based on prokaryotic studies; research on dissimilatory NO_3_^-^ reduction by eukaryotes and its quantitative impact on marine N-cycling is still in its infancy. The seminal work on marine eukaryotes that dissimilatorily reduce NO_3_^-^ was done by Risgaard-Petersen et al. [18]. The authors discovered that the foraminifer *Globobulimina pseudospinescens* store NO_3_^-^ in large quantities, and use it for complete denitrification under anoxic conditions. In following studies on diverse benthic foraminifera and a few gromiida from different benthic habitats, denitrification capacity was found for all analyzed species that contained intracellular NO_3_^-^ [19-21]. In some foraminifera, denitrification is likely carried out by endobionts [22]. The storage of NO_3_^-^ might be a prerequisite for eukaryotes that can switch between O_2_ and NO_3_^-^ respiration, because NO_3_^-^ can be taken up and stored under favorable, oxic conditions for the usage in habitats that can be temporarily exposed to anoxic conditions. 

So far, all marine eukaryotes that have been found to dissimilatorily reduce NO_3_^-^ originate from benthic habitats in which anoxic conditions are common. This study addresses the response of the pelagic, NO_3_^-^-storing diatom *Thalassiosira weissflogii* to darkness and anoxia with respect to dissimilatory NO_3_^-^ reduction and stability of photopigments. Pelagic diatoms may be exposed to anoxic or hypoxic conditions in algal blooms, if O_2_ consumption by the community exceeds O_2_ production, e.g. at night. After the blooms, diatoms might also pass through the anoxic water layers of OMZs [12] and further sink towards the seafloor onto dark/ anoxic sediments [2,23]. The occurrence and viability of *Thalassiosira* species in marine sediments is indeed well known (e.g. 24-26). We hypothesize that a survival mechanism must exist that is energized by dissimilatory NO_3_^-^ reduction. To test this hypothesis, we cultured an axenic *T. weissflogii* strain and followed the consumption of intracellularly stored ^15^NO_3_^-^ after a sudden shift to dark/anoxic conditions as well as the production of end products, by-products, and intermediates of denitrification and DNRA. We further investigated the stability of photopigments after exposure to darkness and anoxia as an indicator of the dark survival potential of *T. weissflogii*.

## Materials and Methods

### Strain and Cultivation

An axenic strain of the marine pelagic diatom *T. weissflogii* (CCMP 1336) was obtained from the Provasoli-Guillard National Center for Marine Algae and Microbiota (NCMA; formerly CCMP). The diatoms were cultured in F/2 medium plus silicate [27] prepared with filtered (0.45 μm) and autoclaved North Sea seawater (salinity 35). The cultivation temperature was 15°C, the light:dark cycle was 10:14 h, and the light intensity was 160 μmol photons m^−2^ s^−1^. *T. weissflogii* was frequently checked for possible contaminations with bacteria by careful phase-contrast microscopy and by plating out subsamples of the cultures on nutrient agar plates. Additionally, all *T. weissflogii* cultures used in the experiments were checked by DAPI staining of cell suspensions immobilized on polycarbonate membrane filters (0.2 μm; Osmonics). A contamination of the diatom strain with prokaryotes was never detected.

### Consumption and Production of Inorganic N-compounds in Dark/Anoxic versus Light/Oxic Conditions

The time courses of intracellular NO_3_^-^ concentrations in *T. weissflogii*, and NO_3_^-^ and NH_4_^+^ concentrations in the growth medium under dark/anoxic versus light/oxic conditions were followed in a non-labeling experiment and a ^15^N-stable isotope labeling experiment (see below). For the non-labeling experiment, the cells were washed with sterile NaCl (salinity 35) and centrifuged (10 min at 1000*g*) three times to remove NO_3_^-^ from the medium, and transferred into NO_3_^-^-free artificial seawater. The cell number was determined (see below), and the experiment was started by dividing the culture for (a) the dark/anoxic incubation, and (b) the light/oxic control. For the dark/anoxic incubation, 20 mL of the diatom suspension was transferred into a dark serum bottle (wrapped in aluminum foil), flushed with N_2_ for 20 min to remove O_2_, sealed with a gas-tight rubber stopper, and incubated at 15°C. For the light/oxic control, the culture was kept under light/oxic culture conditions (see above). At time intervals of 0, 1, 2, 3, 4, 5, 6, and 7 h, 2 mL diatom suspension each was taken and transferred into a sample tube for centrifugation (10 min at 1000*g*). To assure anoxia, the dark serum bottle was flushed with N_2_ after each sampling for 2 min. NO_3_^-^ and (non-labeled) NH_4_^+^ were determined in the cell-free supernatant and the diatom pellet was used for measurements of intracellular NO_3_^-^.

NO_3_^-^ was measured with an NO_x_ analyzer connected to a reaction chamber (CLD 66s plus a Liquid NO Setup; EcoPhysics). In the reaction chamber, acidified VCl_3_ (0.1 M) reduces NO_3_^-^ plus NO_2_^-^ to NO at 90°C, which is then measured by a chemiluminescence detector [28]. If not noted differently, the results of the NO_3_^-^ plus NO_2_^-^ analyses are reported as NO_3_^-^ concentrations throughout, because NO_2_^-^ concentrations were << NO_3_^-^ concentrations. For intracellular NO_3_^-^ measurements, the diatom pellet was directly injected into the reaction chamber where cells burst and release the stored NO_3_^-^. Intracellular NO_3_^-^ concentrations were calculated from the difference of NO_3_^-^ concentrations in the medium and the cell pellet, the cell numbers in the pellet, and the average cell volume of 1.22 pL [8]. Cell numbers were counted in a Fuchs-Rosenthal counting chamber with phase-contrast microscopy at 400× magnification. The total cell number in the medium was used to calculate the total intracellular NO_3_^-^ concentration per volume of medium from the cell-specific intracellular NO_3_^-^ concentration. Ammonium was measured by photometric absorbance determination at λ = 640 nm with a Genesys 10S spectrophotometer (Thermo Scientific; USA) following the sodium-nitroprusside-catalyzed reaction of NH_4_^+^ ions with salicylate and hypochlorite [29].

### Final Products of Dissimilatory Nitrate Reduction

The time courses of intracellular NO_3_^-^ consumption and the possible products of dissimilatory NO_3_^-^ reduction, i.e. NH_4_^+^ for DNRA and N_2_ for complete denitrification, were investigated with a ^15^N-stable isotope labeling experiment. Prior to the experiment, the (non-labeled) intracellular NO_3_^-^ pools of *T. weissflogii* were depleted by a starvation procedure. The cells were separated from the NO_3_^-^-containing culture medium via gentle centrifugation (10 min at 1000*g*), transferred into NO_3_^-^-free artificial seawater [30], and exposed to dark/anoxic conditions for six days. After this pre-incubation, (non-labeled) intracellular NO_3_^-^ had been completely consumed. For the subsequent storage of intracellular ^15^N-labeled NO_3_^-^ (98 atom%; Cambridge Isotope Laboratories), the NO_3_^-^-starved cells were harvested, re-inoculated into sterile, ^15^NO_3_^-^-containing F/2 medium plus silicate in artificial seawater, and cultured under optimal growth conditions for three days (see above). The cells were then washed via gentle centrifugation with sterile NO_3_^-^-free artificial seawater (salinity 35; 10 min at 1000*g*) to remove ^15^NO_3_^-^ from the medium, and transferred into NO_3_^-^-free artificial seawater enriched with 200 μM Na-acetate and 25 μM non-labeled NH_4_^+^. Thus, the only NO_3_^-^ source during the ^15^N-stable isotope labeling experiment was ^15^NO_3_^-^ stored intracellularly by the diatoms. The cell density was obtained and the experiment was started by dividing the culture for (a) the dark/anoxic incubation, and (b) the light/oxic control. For the dark/anoxic incubation, ca. 200 mL of the diatom suspension was transferred into a dark bottle (wrapped in aluminum foil) and flushed with He for 30 min to remove O_2_ and then transferred into 24 replicate 6 mL Labco-exetainers® wrapped in aluminum foil. At time intervals of 1, 2, 3, 4, 5, 6, 8, and 10 h, a He headspace of 3 mL was set in three Labco-exetainers® each, and the diatom cells in the remaining 3 mL were killed with 100 µL ZnCl_2_ (50%). The Labco-exetainers® were stored upside down at room temperature until measurement of ^15^N-labeled N_2_ by gas chromatography-isotope ratio mass spectrometry (GC-IRMS, VG Optima; Isotech). The cell suspension collected during setting the headspace was filled into 15-mL tubes and centrifuged (10 min at 1000*g*). Part of the cell-free supernatant was used for immediately measuring the extracellular NO_3_^-^ concentrations, while the pellet was used for intracellular NO_3_^-^ determination (see above). Further, 1 mL cell-free supernatant was frozen at -20°C until ^15^NH_4_^+^ analysis using the hypobromite assay [31], followed by N_2_-^15^N analysis using GC-IRMS. The hypobromite assay actually measures the sum of ^15^NH_4_^+^ and ^15^N-labeled volatile N compounds such as methyl amines [32]. For the light/oxic control, the culture was kept under light/oxic conditions, and at time intervals of 0, 1, 2, 5, and 10 h, 3 mL cell material each was taken and processed exactly like the material that was obtained during setting the headspace in the dark/anoxic treatment. The sample collected at time point zero was used for both, the dark/anoxic incubation and the light/oxic control.

### Intermediates and By-Products of Dissimilatory Nitrate Reduction

The time courses of N_2_O and NO_2_^-^ as possible intermediates or by-products of dissimilatory NO_3_^-^ reduction were measured during the ^15^N-stable isotope labeling experiment. Nitrous oxide was measured in the headspace of the Labco-exetainers® from the dark/anoxic incubation experiment after N_2_-^15^N analysis had been completed (see above; the gas volume removed for N_2_-^15^N measurements was taken into account for the subsequent calculation of N_2_O concentrations), using a GC 7890 (Agilent Technologies) equipped with a CP-PoraPLOT Q column and a ^63^Ni electron capture detector. Nitrite was determined in the supernatant of the medium from the dark/anoxic and the light/oxic incubation with an NO_x_ analyzer as described for the NO_3_^-^ determination, except that the reaction chamber contained acidified NaI (2 M) that reduces NO_2_^-^ to NO at 20°C.

### Degradation of Photopigments in Response to Dark/Anoxic Conditions

Chlorophyll a and fucoxanthin were determined in cultures of *T. weissflogii* that were first exposed to favorable growth conditions (i.e. with light and O_2_; time 0) and then to dark/anoxic conditions for a time period of 46 weeks. To adjust dark/anoxic conditions, diatom cultures were transferred into gas-tight, dark bottles (wrapped in aluminum foil), flushed with N_2_ for 30 min and kept at 15°C until sampling. At each time point, 2 mL of the cell suspension was taken in 3 replicates and cell numbers were counted. The samples were freeze-dried for 2 days and 5 mL ice-cold acetone was added for extraction of photopigments. After vigorous mixing and sonication for 5 min, the samples were left over night at -20°C, mixed again, and centrifuged for 5 min at 3000*g*. The supernatants were filtered (Acrodisc^®^ CR 4 mm, 0.45 µm Versapor^®^; Gelman Laboratory) and the extracted photopigments were separated by means of HPLC (Waters 2695; U.S.A.) and analyzed by a photodiode array detector (Waters 996; U.S.A.) as described in Stief et al. [26]. In the chromatograms, chlorophyll *a* and fucoxanthin were identified according to their specific retention time and absorption spectra and the respective peaks were integrated with the Millenium®32 software (Waters, U.S.A.). Calibrations were made with serial dilutions of chlorophyll *a* and fucoxanthin stock solutions (DHI, Denmark). All procedures were made under dark conditions and using HPLC-grade chemicals.

## Results and Discussion

### Dissimilatory Nitrate Reduction to Ammonium by *T. weissflogii*

**Figure 1:**
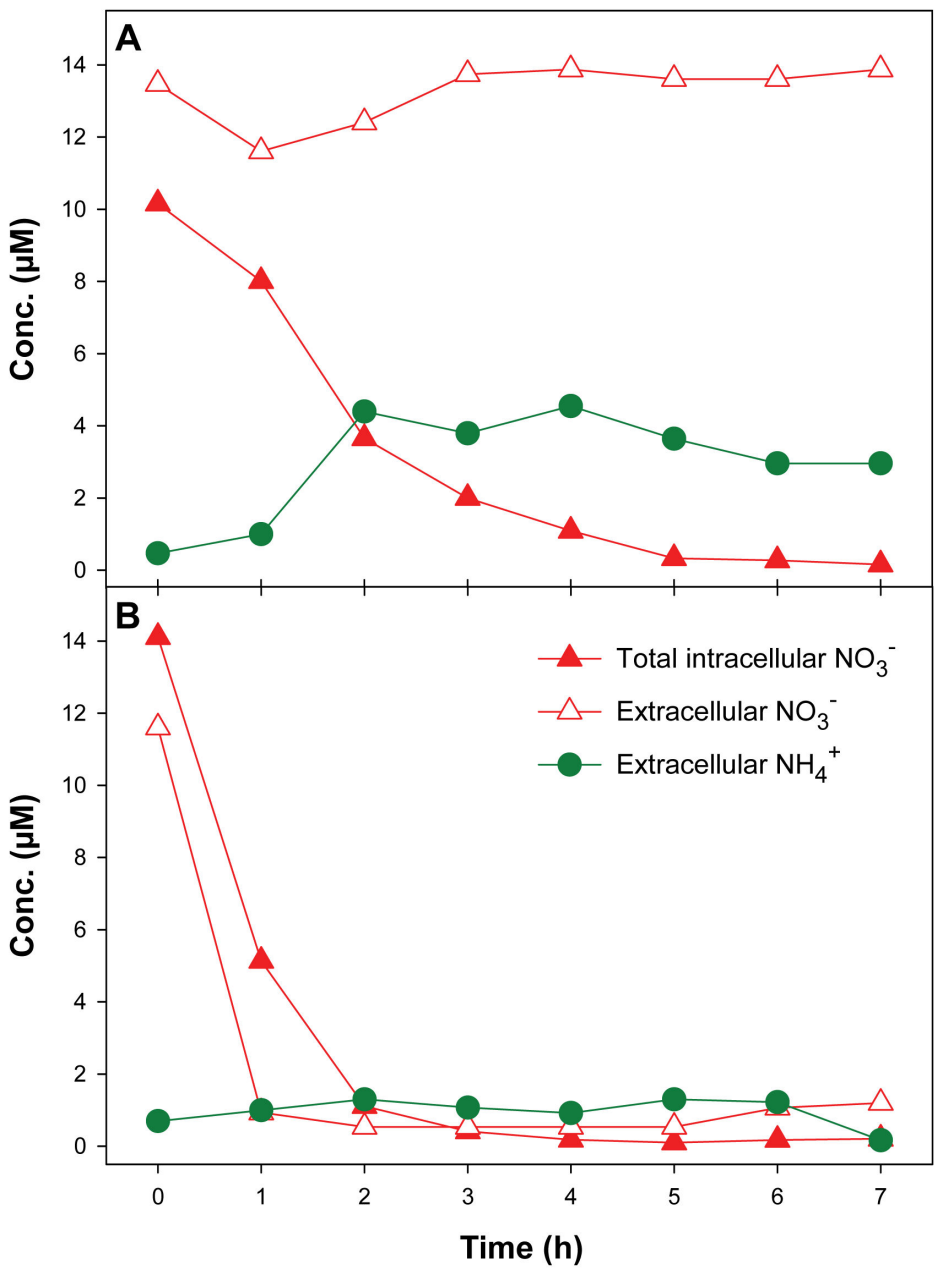
Non-labeling experiment. Time courses of total intracellular NO_3_^-^ (expressed in μmol L^-1^ of growth medium), and extracellular NO_3_^-^ and NH_4_^+^ concentrations in an axenic *T. weissflogii* culture in response to (A) dark/anoxic conditions, and (B) light/oxic conditions. Dark/anoxic conditions were initiated at time point 0.

**Figure 2:**
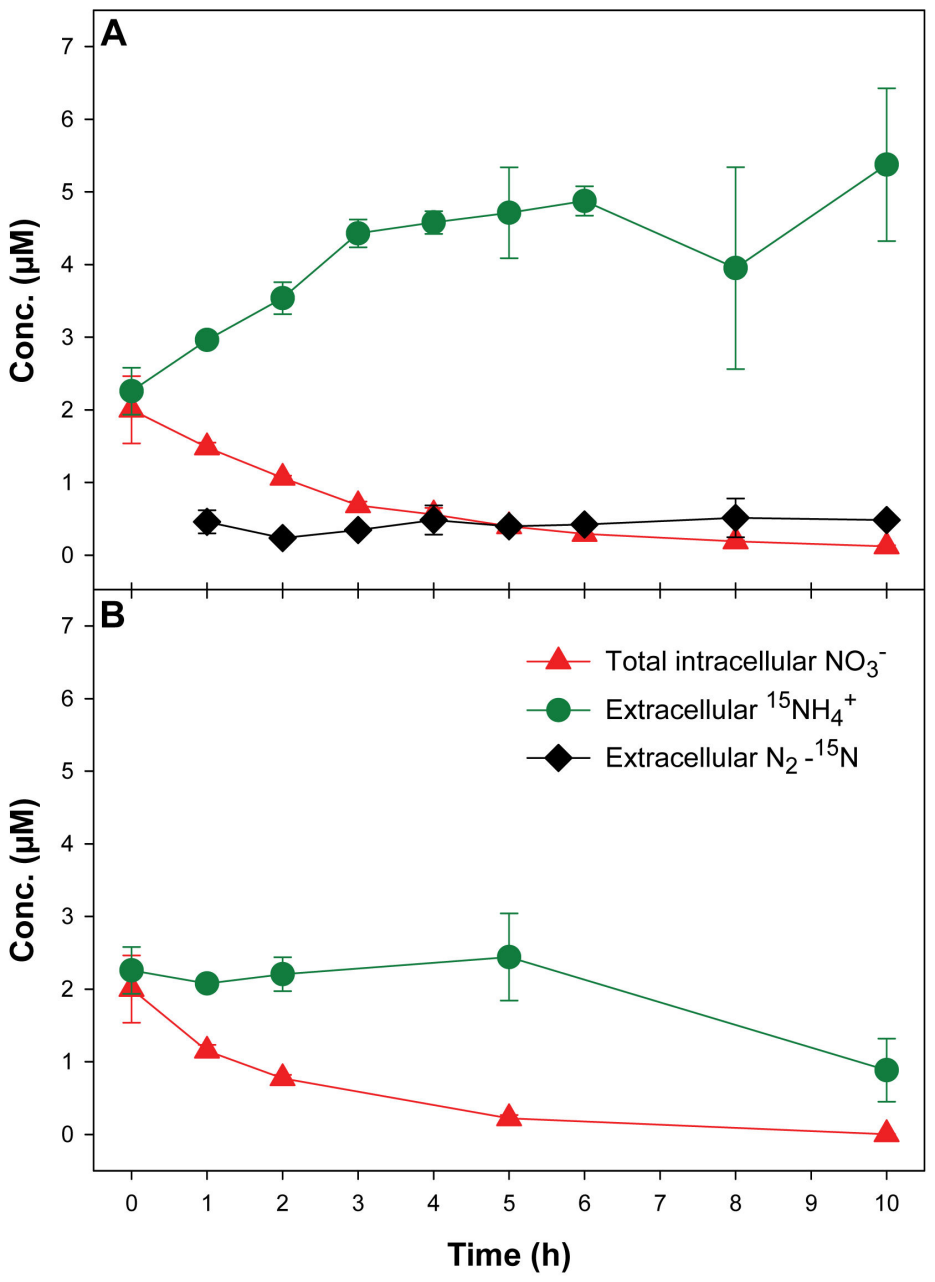
^15^N-stable isotope labeling experiment. Time courses of total intracellular ^15^NO_3_^-^ (expressed in μmol L^-1^ of growth medium), and extracellular ^15^NH_4_^+^ and N_2_-^15^N concentrations in an axenic *T. weissflogii* culture in response to (A) dark/anoxic conditions, and (B) light/oxic conditions. Dark/anoxic conditions were initiated directly after time point 0. Some error bars, which indicate standard deviation (n=3), are smaller than the symbols. The NO_3_^-^ was measured with a non-labeling sensitive technique and is consequently not plotted as ^15^NO_3_^-^. However, the only NO_3_^-^ source in the experiment was ^15^NO_3_^-^ (see Materials and Methods).

Our results strongly indicate that the ubiquitous pelagic diatom *T. weissflogii* is able to perform DNRA, similar to the benthic diatom *Amphora coffeaeformis*, which was the first phototrophic eukaryote shown to dissimilatorily reduce NO_3_^-^ under dark/anoxic conditions [8]. Consumption of intracellular NO_3_^-^ and simultaneous production of NH_4_^+^ in response to dark/anoxic vs. light/oxic conditions have been followed in two separate experiments: (a) a non-labeling experiment in which NH_4_^+^ was measured photometrically (Figure 1) and (b) a ^15^N-stable isotope labeling experiment (Figure 2). In both experiments, the rapid consumption of intracellular NO_3_^-^ and ^15^NO_3_^-^ by *T. weissflogii* was accompanied by the production and release of NH_4_^+^ and ^15^NH_4_^+^, respectively, only under dark/anoxic conditions, but not in the presence of light and O_2_ (Figures 1,2). In the ^15^N-stable isotope labeling experiment, the initial ^15^NH_4_^+^ concentration was 2 µM because the hypobromite assay actually measures the sum of ^15^NH_4_^+^ and ^15^N-labeled volatile N compounds such as methyl amines [32]. The concentration of NO_3_^-^ in the medium, i.e. extracellular NO_3_^-^, only decreased under light/oxic conditions, but remained constant after exposure to dark/anoxic conditions (Figure 1). This constant (and not increasing) extracellular NO_3_^-^ concentration indicates that the intracellular NO_3_^-^ (expressed in μmol L^-1^ of growth medium) was indeed consumed by *T. weissflogii* rather than released from the cells into the medium. Intracellular NO_3_^-^ was also consumed under light/oxic conditions, even at a higher rate than under dark/anoxic conditions (Tables 1,2), most probably because NO_3_^-^ was used for assimilation by photosynthetically active diatoms [33-35]. For N-assimilation, NO_3_^-^ is also reduced to NH_4_^+^, but NH_4_^+^ is not released from the cells.

**Table 1:** Cell-specific consumption (neg. values) rates of NO_3_^-^ by axenic *T. weissflogii* cultures in response to different experimental conditions for the non-labeling experiment.

**Experimental conditions**	**Time interval (h)**	**NO_3_^-^ (fmol N cell^-1^ h^-1^)**
**Dark/anoxic**	0 – 2	-7.47
	2 - 7	-1.54
**Light/oxic**	0 – 2	-12.38
	2 - 7	-0.29

Rates were calculated from the time course of NO_3_^-^ presented in Figure 1 for linear concentration changes in the given time intervals. Cell densities were 435 cells µL^-1^ for dark/anoxic conditions, and 525 cells µL^-1^ for light/oxic conditions; the initial intracellular NO_3_^-^ concentration was 20 mM.

**Table 2:** Cell-specific consumption (neg. values) and production (pos. values) rates of N compounds by axenic *T. weissflogii* cultures in response to different experimental conditions for the ^15^N-stable isotope labeling experiment.

**Experimental conditions**	**Time interval (h)**	**NO_3_^-^ (fmol N cell^-1^ h^-1^)**	**^15^NH_4_^+^ (fmol N cell^-1^ h^-1^)**	**N_2_O (fmol N cell^-1^ h^-1^)**	**NO_2_^-^ (fmol N cell^-1^ h^-1^)**
**Dark/anoxic**	0 – 2	-1.150 (±0.253)	+1.571 (±0.212)	+0.007 (±0.002)*	+0.049 (±0.047)
	2 - 10	-0.261 (±0.028)	+0.313 (±0.159)	+0.002 (±0.001)	+0.042 (±0.006)
**Light/oxic**	0 – 2	-1.508 (±0.283)	-0.066 (±0.233)	ND	ND
	2 - 10	-0.224 (±0.035)	-0.442 (±0.141)	ND	ND

**Figure 3:**
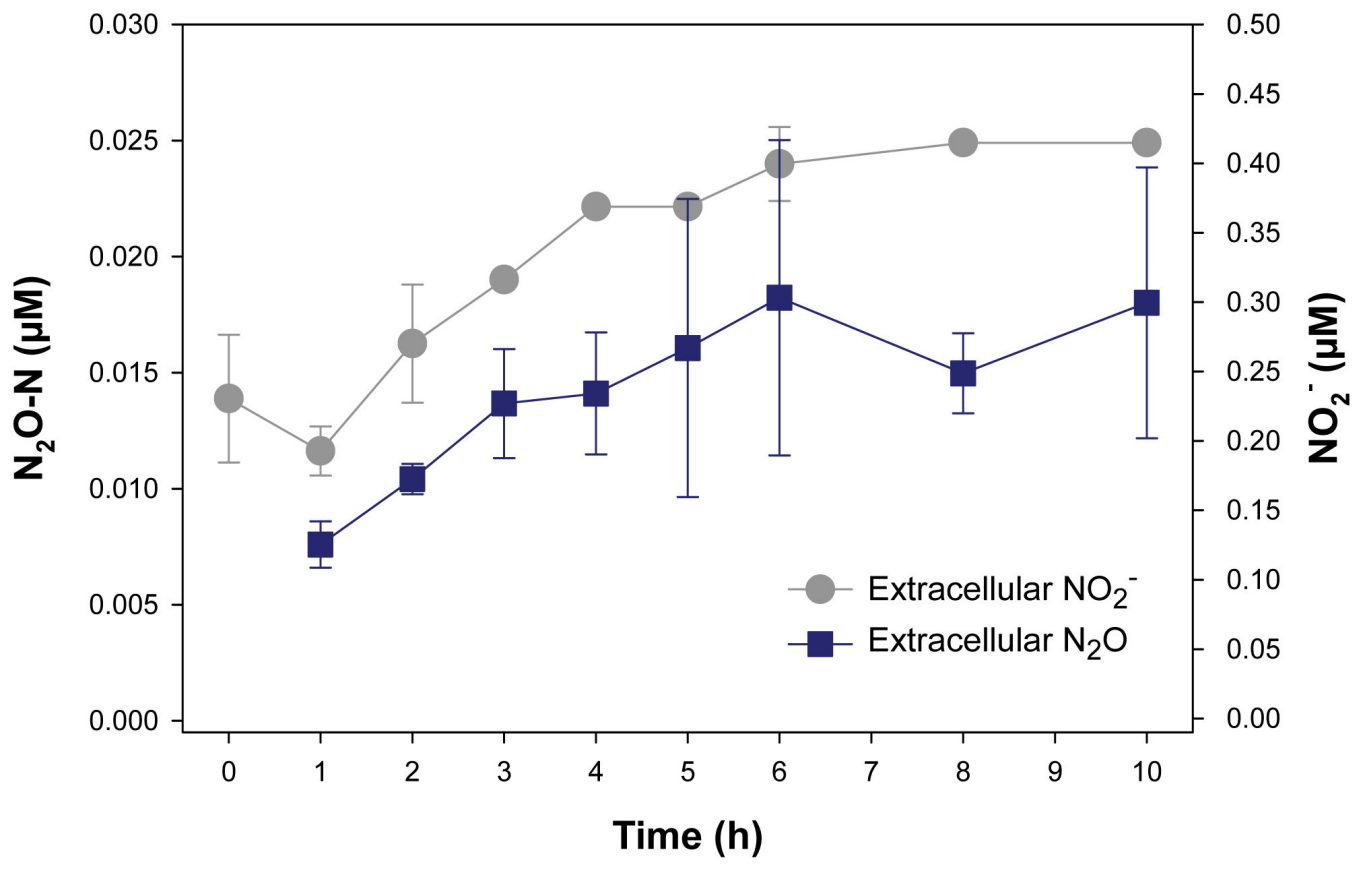
Time courses of extracellular N_2_O and NO_2_^-^ concentrations in an axenic *T. weissflogii* culture in response to dark/anoxic conditions. Dark/anoxic conditions were initiated directly after time point 0. Nitrous oxide and NO_2_^-^ concentrations were measured in samples from the ^15^N-stable isotope labeling experiment using techniques that do not distinguish between ^14^N- and ^15^N-labeled forms of N_2_O and NO_2_^-^. Some of the error bars, which indicate standard deviation (n=3), are smaller than the symbols.

Rates were calculated from the time course of N compounds presented in Figures 2 and 3 for linear concentration changes in the given time intervals. Cell densities were 407 cells µL^-1^; the initial intracellular NO_3_^-^ concentration was 4 mM.

Means (±SE) for n=3 are shown; ND: not determined; *Rate calculated for 1 - 2 h only.

In the absence of O_2_, intracellular NO_3_^-^ can be used for dissimilation by sulfur bacteria [36-39] and only a few unicellular eukaryotes and fungi (e.g. [8,18,20,40,41]). The ubiquitous diatom *T. weissflogii* can now be added to the short list of eukaryotes that dissimilatorily reduce NO_3_^-^. Notably, *T. weissflogii* is the first marine pelagic eukaryote shown to have an anaerobic NO_3_^-^ metabolism, whereas all known eukaryotic NO_3_^-^ reducers thrive in stratified waters, sediments and soils in which anoxic conditions occur in subsurface layers. So far it is not known, whether DNRA in *T. weissflogii* is respiratory or fermentative. Briefly, in respiratory DNRA, ATP is generated by an electrochemical proton potential across a cell membrane, at which electrons are transferred from the donor to the acceptor NO_3_^-^, and in fermentative DNRA, ATP is generated by substrate-level phosphorylation [16,42-44]. In prokaryotes, the electron donor and acceptor for respiratory DNRA usually originate from an external source and not from cell metabolism, but may be either organic or inorganic, whereas the electron donor in fermentative DNRA is usually organic [45]. So far, the electron donor used by diatoms for DNRA is not known. In our labeling experiment, acetate was added as a potential electron donor. However, it needs to be further investigated, if *T. weissflogii* can perform DNRA also with intracellularly stored electron donors, like polysaccharides (e.g. chrysolaminarin), and if the external supply of acetate indeed influences the rate of DNRA.

Our experiments revealed that the rate of NO_3_^-^ consumption after exposure to dark/anoxic conditions depends on the concentration of intracellularly stored NO_3_^-^. In the non-labeling experiment, the initial intracellular NO_3_^-^ concentration was 20 mM, and in the labeling experiment only 4 mM, resulting in a 6 times lower rate of NO_3_^-^ consumption (Tables 1,2). In the labeling experiment, the production of ^15^NH_4_^+^ (plus N_2_O and NO_2_^-^) by *T. weissflogii* balanced the consumption of intracellular ^15^NO_3_^-^ within the bounds of accuracy (Figure 2; Table 2), whereas in the non-labeling experiment, the net production of NH_4_^+^ did not balance the consumption of intracellular NO_3_^-^ (Figure 1). On average, less than half of the NO_3_^-^ was found back as NH_4_^+^ in the culture medium; further, the NH_4_^+^ concentration first increased and then decreased slightly with time (Figure 1). This decrease of the NH_4_^+^ concentration in the non-labeling experiment is explained by an uptake of NH_4_^+^ by *T. weissflogii* under dark/anoxic conditions that has also been confirmed in other experiments (data not shown). A dark NH_4_^+^ uptake and assimilation, respectively, is generally known for phytoplankton [34,46] and was recently also confirmed by gene expression analysis in *Thalassiosira pseudonana* [47]. This dark NH_4_^+^ uptake is not apparent in the labeling experiment, because the addition of non-labeled NH_4_^+^ as background concentration (see Materials and Methods) obscures the putative uptake of ^15^NH_4_^+^. However, under the light/oxic conditions of the labeling experiment, ^15^NH_4_^+^ also decreased after 5 h because the (non-labeled) background NH_4_^+^ was completely taken up (data not shown). The labeling approach did not reveal a production of N_2_-^15^N by *T. weissflogii* (Figure 2), which further supports that DNRA and not denitrification is used as a dissimilatory NO_3_^-^ reduction pathway by *T. weissflogii*.

### Release of Nitrous Oxide and Nitrite during Nitrate Dissimilation

The production of N_2_O and NO_2_^-^ in response to dark/anoxic conditions has been followed during the ^15^N-stable isotope labeling experiment (Figure 3). Both, N_2_O and NO_2_^-^ were produced and released from the cells in the same time pattern that has been observed for ^15^NH_4_^+^, and their production apparently mirrors the consumption of intracellular NO_3_^-^ (Figures 1,2,3). However, the production rates of N_2_O and NO_2_^-^ were about 1000 and 100 times, respectively, lower than the production rate of ^15^NH_4_^+^ (Table 2). Thus, N_2_O and NO_2_^-^ are not final products of dissimilatory NO_3_^-^ reduction, but the congruent time patterns indicate that *T. weissflogii* releases N_2_O and NO_2_^-^ as by-product and intermediate, respectively, of DNRA. In prokaryotes, N_2_O is a well-known by-product and intermediate of nitrification and denitrification, respectively, and there are some indications that N_2_O is also released as a by-product of DNRA, which might have been overseen in some organisms [16,48]. In higher plants, N_2_O is emitted from leaves by plant NO_3_^-^ assimilation, strictly speaking during photoassimilation of NO_2_^-^ in the chloroplast [49]. Recently, N_2_O production was also found in axenic, illuminated cultures of the green algae *Chlorella vulgaris* [50]. A release of N_2_O by phototrophic eukaryotes under darkness and anoxia has to our knowledge not been documented so far. Even though the rate of N_2_O released by *T. weissflogii* during DNRA might seem low (see above, Table 2), this finding can be of environmental importance because diatoms are highly abundant in the world’s oceans (e.g. [3,51]), hypoxic and anoxic marine environments are spreading [52], and N_2_O is a particularly strong greenhouse gas [53]. The production of N_2_O under dark/anoxic conditions has recently also been confirmed for the benthic diatom *A. coffeaeformis* [8], and it might be worth to screen other benthic and pelagic diatom species for N_2_O emission under these conditions.

The NO_2_^-^ release during DNRA by *T. weissflogii* could be due to cell leakage or excretion that is frequently observed in marine phytoplankton, including diatoms [54,55], but has not been linked to a response of phytoplankton to darkness and anoxia so far. The observed NO_2_^-^ release might be supported by a slightly higher rate of NO_3_^-^ reduction than NO_2_^-^ reduction throughout the incubation. Further, there might be a time delay in NO_2_^-^ reduction to NH_4_^+^ because of constitutive expression of the NO_3_^-^-reductase gene, whereas the (dissimilatory) NO_2_^-^-reductase gene first needs to be induced by the production of NO_2_^-^. *T. weissflogii* is not able to take up the released NO_2_^-^ again under dark/anoxic conditions, which is indicated by the observation that the medium NO_2_^-^ concentration is not decreasing during the incubation (Figure 3). Additionally, intracellular NO_2_^-^ storage does not occur in *T. weissflogii* (data not shown), probably because of the toxic effects of NO_2_^-^ [54].

### Slow Degradation of Photopigments in Darkness and Anoxia

**Figure 4:**
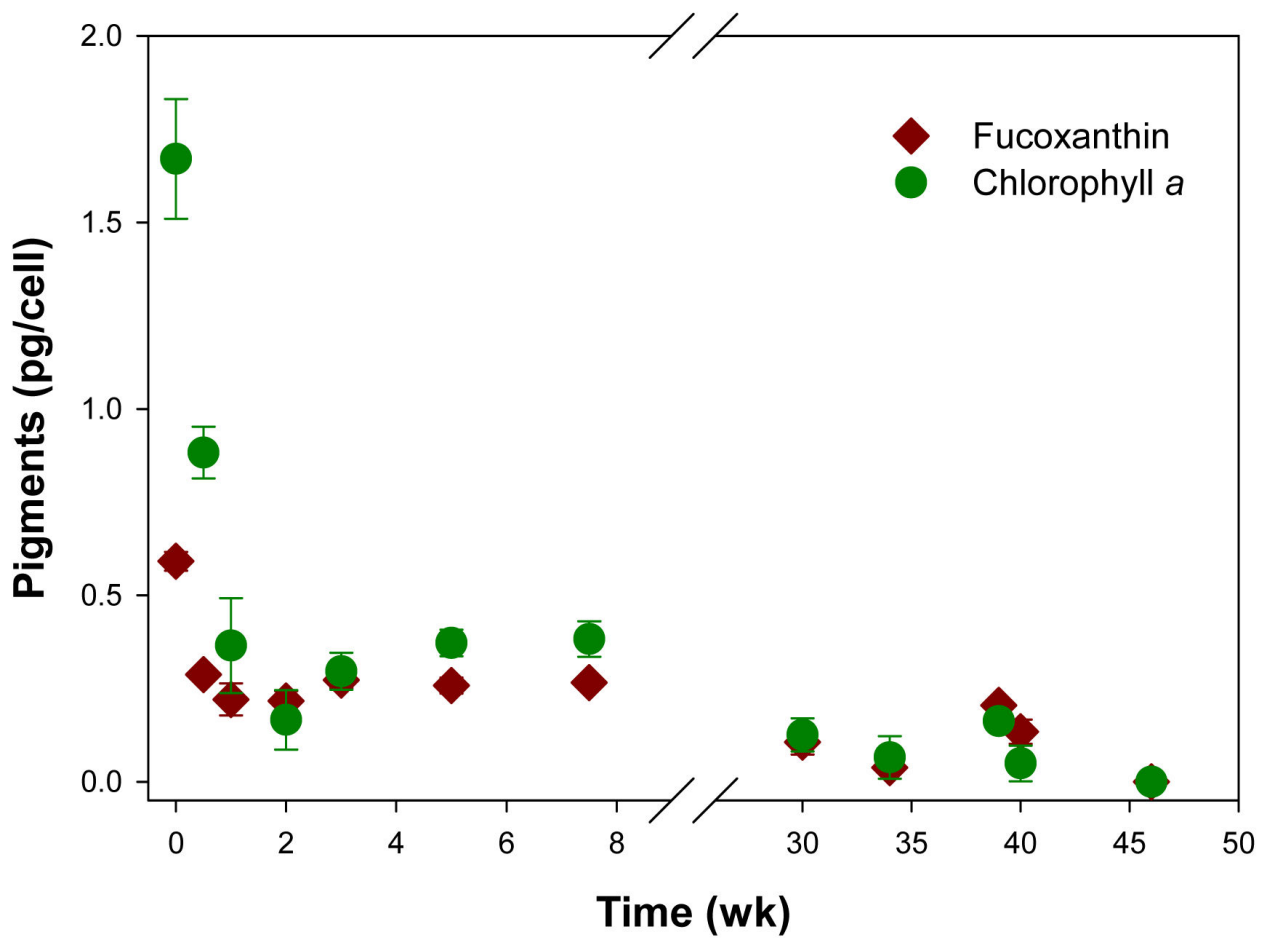
Time courses of the intracellular chlorophyll *a* and fucoxanthin concentrations in an axenic *T. weissflogii* culture in response to dark/anoxic conditions. Dark/anoxic conditions were initiated directly after time point 0. Some of the error bars, which indicate standard deviation (n=3), are smaller than the symbols.

To estimate how long *T. weissflogii* cells retain the ability to operate photosynthesis after exposure dark/anoxic conditions, the fate of the photopigments chlorophyll a and fucoxanthin was followed. Notably, the degradation of the photopigments did not temporally coincide with DNRA by *T. weissflogii* in response to dark/anoxic conditions. While DNRA activity peaked during the first few hours of dark/anoxic conditions, the major decrease in cellular photopigment contents occurred during the first 3 days (Figures 2,4). After one week of dark/anoxic incubation, the cellular pigment contents had reached a low, but constant level that was maintained for at least 7.5 weeks (Figure 4). These observations are in good agreement with the hypothesis that diatoms use DNRA to enter a resting stage with low metabolic activity, and that *T. weissflogii* was found to survive at least for 6 weeks after adjusting them to dark/anoxic conditions [8]. Diatoms are known to start photosynthesis and growth very fast after (re)adjusting them to favorable growth conditions, i.e. light and fresh growth medium, even after extended periods of darkness [24,56,57]. To maintain at least low cellular contents of photopigments must be a prerequisite for this. Our experimental design, i.e. that no O_2_ and prokaryotes were present in the *T. weissflogii* culture, further led to a decreased rate of degradation, as O_2_-dependent pigment alteration and grazing-induced cell disruption could not occur [58]. Interestingly, the chloroplasts of *T. weissflogii* cells showed an autofluorescence even after more than 1 year under dark/anoxic conditions (pictures not shown), which might originate from photopigment degradation products that are still poorly understood [59].

### Ecological and Evolutionary Perspectives

After the benthic diatom *A. coffeaeformis* was discovered as the first photothrophic eukaryote that dissimilatorily reduces NO_3_^-^, it was interesting to ask whether this metabolism also occurs in pelagic diatoms: and indeed, we found *T. weissflogii* as the so far only marine pelagic eukaryote showing this metabolic trait. The respiration of NO_3_^-^ by diatoms might be widespread in marine ecosystems and could have so far overseen implications on the marine N-cycle. For benthic foraminifera, Piña-Ochoa et al. [20] calculated a contribution for the removal of fixed N from marine ecosystems that may be equally important to bacterial denitrification in the seafloor. DNRA will not directly remove fixed N, but in anoxic or hypoxic environments, the produced NH_4_^+^ can serve as electron donor for anammox that might be especially important in OMZs with high abundances of anammox bacteria [60]. Further research on NO_3_^-^ respiration by diatoms might also reveal that certain species are capable of other pathways than DNRA, like denitrification as shown for foraminifera [18,20]. Additionally, the exact ambient O_2_ concentration in the (micro)environment of the diatoms may trigger different dissimilatory NO_3_^-^ reduction pathways as known from fungi [45].

To date, genes involved in dissimilatory NO_3_^-^ reduction have not been identified in NO_3_^-^-respiring diatoms, foraminifera or gromidii. In contrast, several functional genes have been identified in the denitrifying fungus *Fusarium oxysporum*: a copper-containing NO_2_^-^ reductase (*nirK*) and a nitric oxide reductase (P450nor) have been sequenced and characterized [41,61]. Intriguingly, NO_3_^-^-respiring fungi may use enzymes that are normally involved in assimilatory NO_3_^-^ reduction in a dissimilatory mode instead [62]. This could also hold true for diatoms. Assimilatory NO_3_^-^ reductases, multiple transporters for NO_3_^-^, and components of a NO_3_^-^-sensing system have only recently been discovered in diatom genomes [63,64]. First insights into diatom genomes and the ensuing ecophysiological studies revealed a fascinating evolutionary history of diatoms. An unexpected combination of genes by endosymbiotic gene transfer from two secondary endosymbionts to the exosymbiont nucleus, and also horizontal gene transfer led to several additional inclusions from Bacteria and Archaea genomes [63-66]. The diverse assortment of genes results in novel biochemical pathways like the urea cycle [63,65,67-71] that formerly was not known for photosynthetic organisms and congruously makes diatoms for Armbrust et al. [51] to be neither plants nor animals. Further work on diatom genomes could lead to the identification of functional genes involved in dissimilatory NO_3_^-^ reduction. This would not only convey genetic evidence of dissimilatory NO_3_^-^ reduction by eukaryotes, but would also provide genetic markers for the cultivation-independent detection of so far unrecognized dissimilatorily NO_3_^-^ reducing diatoms directly in the environment.
